# Compliance and toxicity of adjuvant CMF in elderly breast cancer patients: a single-center experience

**DOI:** 10.1186/1471-2407-5-30

**Published:** 2005-03-24

**Authors:** Ermelinda De Maio, Adriano Gravina, Carmen Pacilio, Gerardo Amabile, Vincenzo Labonia, Gabriella Landi, Francesco Nuzzo, Emanuela Rossi, Giuseppe D'Aiuto, Immacolata Capasso, Massimo Rinaldo, Brunello Morrica, Massimo Elmo, Massimo Di Maio, Francesco Perrone, Andrea de Matteis

**Affiliations:** 1 Clinical Trials Unit, National Cancer Institute, Naples, Italy; 2 Division of Medical Oncology C, National Cancer Institute, Naples, Italy; 3 Division of Surgical Oncology A, National Cancer Institute, Naples, Italy; 4 Division of Radiotherapy, National Cancer Institute of Naples, Italy

## Abstract

**Background:**

Few data are available on compliance and safety of adjuvant chemotherapy when indicated in elderly breast cancer patients; CMF (cyclophosphamide, methotrexate, fluorouracil) can be reasonably considered the most widely accepted standard of treatment.

**Methods:**

We retrospectively reviewed compliance and safety of adjuvant CMF in patients older than 60. The treatment was indicated if patients had no severe comorbidity, a high-risk of recurrence, and were younger than 75. Toxicity was coded by NCI-CTC. Toxicity and compliance were compared between two age subgroups (<65, ≥ 65) by Fisher exact test and exact Wilcoxon rank-sum test.

**Results:**

From March 1991 to March 2002, 180 patients were identified, 100 older than 60 and younger than 65, and 80 aged 65 or older. Febrile neutropenia was more frequent among older patients (p = 0.05). Leukopenia, neutropenia, nausea, cardiac toxicity and thrombophlebitis tended to be more frequent or severe among elderlies, while mucositis tended to be more evident among younger patients, all not significantly. Almost one half (47%) of the older patients receiving concomitant radiotherapy experienced grade 3–4 haematological toxicity. Compliance was similar in the two groups, with 6 cycles administered in 86% and 79%, day-8 chemotherapy omitted at least once in 36% and 39%, dose reduction in 27% and 38%, prolonged treatment duration (≥ 29 weeks) in 10% and 11% and need of G-CSF in 9% and 18%, among younger and older patients, respectively.

**Conclusion:**

Our data show that, in a highly selected population of patients 65 or more years old, CMF is as feasible as in patients older than 60 and younger than 65, but with a relevant burden of toxicity. We suggest that prospective trials in elderly patients testing less toxic treatment schemes are mandatory before indicating adjuvant chemotherapy to all elderly patients with significant risk of breast cancer recurrence.

## Background

Breast cancer is a very frequent malignancy among elderly patients. Its incidence increases with age, up to 80 years of age [1]. Furthermore, there are convincing data from the Surveillance, Epidemiology, and End Result (SEER) Program [2] showing that the proportion of elderly patients with breast cancer has been increasing during the last decades; patients aged 65 or more, indeed, accounted for 37% of breast cancer cases diagnosed in 1973 and for 47% of those diagnosed in 1995.

Nowadays, adjuvant chemotherapy for breast cancer is a standard clinical practice. This strategy has solid bases in the data of the Oxford Overview by the Early Breast Cancer Trialists' Collaborative Group [3] that showed unequivocal benefit of adjuvant chemotherapy and suggested that its positive effect could be additive to that of tamoxifen. Thus, during recent years there has been a trend toward giving chemotherapy to the majority of women with early breast cancer, including the elderly ones if their risk of relapse is high and their clinical conditions are not deteriorated; thus, without considering elderly patients like a special subgroup for which specific recommendations are needed [4-6]. However, data collected in the Overview do not directly demonstrate the usefulness of adjuvant chemotherapy in older patients. Larger number of patients are needed, possibly enrolled into clinical trials specifically planned for elderly women, like the recently published study conducted by the French Adjuvant Study Group [7]. This study, indeed, evaluated the efficacy of the addition of weekly epirubicin to tamoxifen for patients older than 65 with node-positive early breast cancer, finding an advantage in terms of disease-free survival for patients receiving chemotherapy. Studies like this are needed to make the evidence stronger and to overcome selection biases possibly deriving from severe selection of elderly patients entered in clinical trials designed for adult ones. Indeed, it is well known that the proportion of elderly patients enrolled in clinical trials is far more lower than the proportion they represent in population statistics [8], barriers to their enrollment being multidimensional [9].

In clinical practice another problem due to the lack of reliable trials is the choice of which type of chemotherapy can be given to elderly patients. While it is now recognized [6] that two general levels of cytotoxic therapy regimens exist, one less aggressive that includes four courses of doxorubicin and cyclophosphamide or six courses of classical CMF (cyclophosphamide, methotrexate and fluorouracil), and another more aggressive including cyclophosphamide, epirubicin, and fluorouracil [10,11] and experimental associations or sequences with taxanes [12,13], many of these schemes have never been tested in prospective trials in elderly patients. With paucity of experimental data, CMF is felt as the more reasonable standard schedule for such patients [14].

While planning a randomized clinical trial, we reviewed the records of early breast cancer patients who received adjuvant CMF (days 1 and 8 every 4 weeks for a planned number of 6 cycles) at the National Cancer Institute of Naples, Italy, during the last decade, to describe compliance and toxicity in patients 65 or more years old; as a comparative group, we collected data of patients of the immediately lower age cohort (older than 60 and younger than 65).

## Methods

We selected charts of patients older than 60 (i.e.: those diagnosed breast cancer after the 60^th ^birthday) who had received CMF from March 1991 to March 2002, outside clinical trials. All the patients had undergone mastectomy or quadrantectomy plus axillary lymphadenectomy. Patients undergoing conservative surgery were scheduled for radiotherapy of the residual breast with a standard scheme (5000 cGy plus 1000 cGy of boost on the quadrant) that was planned either concomitantly with adjuvant chemotherapy or immediately after the end of it, according to waiting lists or patient's or physician's preference. When radiotherapy was planned concomitantly, it was administered between the 3^rd ^and the 4^th ^cycle of CMF, in a sandwich scheme, producing a 6 weeks prolongation of the overall duration of CMF treatment.

In the period of interest, at our Institution, criteria on which therapeutic decisions regarding adjuvant chemotherapy were taken outside clinical trials varied according to the evolution of international guidelines and the results of the Oxford metanalysis. Overall, we recommended chemotherapy to patients aged less than 75, who had undergone surgical treatment (mastectomy or quadrantectomy, plus axillary lymphnode dissection), with a high risk of recurrence (based on common features like positive nodes, large primary tumor, high grade of malignancy, estrogen-receptor negative), good performance status (ECOG PS 0 or 1), normal function of vital organs (bone marrow, kidney, liver and heart) and no other severe comorbidity contraindicating chemotherapy. As older the patients and as lower the estimate of recurrence risk, the higher was the chance of choosing CMF rather than an anthracycline-containing regimen. Estrogen receptor positive patients, treated with chemotherapy, received tamoxifen after the end of chemotherapy.

For this analysis, we focused on patients aged over 60, who had received CMF according to the following schedule: cyclophosphamide 600 mg/m^2^, methotrexate 40 mg/m^2 ^and fluorouracil 600 mg/m^2^, on days 1 and 8, every 4 weeks, for 6 planned cycles. With this schedule, common supportive care was applied consisting of antiemetic medication with 5HT_3 _antagonist on the days of chemo, antibiotics in case of known bacterial infection associated with granulocyte colony-stimulating factor (G-CSF) in case of febrile neutropenia; G-CSF was also sporadically used in case of grade 4 neutropenia without fever, and prophylactically in cycles following febrile or grade 4 neutropenia.

Pre-chemotherapy evaluation included medical history, physical examination, haematology, serum biochemistry tests, and tumor staging with chest radiography, abdominal ultrasonography, nuclidic bone scan with X-ray of bone hot-spots, ECG and, in selected cases, an evaluation of left ventricular ejection fraction. Toxicity was usually assessed with blood counts preceding each administration of chemotherapy, and complete serology before day 1 administration of chemotherapy, unless clinical need for more frequent control. Reviewing the charts, we codified toxicity according to National Cancer Institute Common Toxicity Criteria (version 2.0, 1998). Toxicity is reported with details of type and grade, including all grades. Anyway, for descriptive purposes we also summarised toxicity into three major groups: haematological (leukopenia, neutropenia, anemia, thrombocytopenia, febrile neutropenia, bleeding, infection), other (including all the other toxicities) and any (including both haematological and other) and we summed up grades 0 to 2 as non severe and grades 3 or 4 as severe.

To analyse compliance, the distribution of several indices was compared between the two age subgroups. A cycle was considered as delivered if day 1 chemotherapy had been administered. Indices used for compliance description were: a) the number of delivered cycles, b) the occurrence of withdrawal of chemotherapy on day 8, c) the occurrence and the percentage of dose-reductions, d) the occurrence of treatment delay that was expressed as relative duration by dividing actual and planned duration (1 means no delay, higher values mean progressively longer delays), e) the need for G-CSF treatment according to previously reported guidelines, and f) the cause of treatment discontinuation if earlier than planned. After description of single indices, a binary index was built, weak compliance being defined as the occurrence of at least one of the following events: less than 6 cycles administered, withdrawal of more than one day-8 treatment, dose reduction in more than 25% of cycles, relative duration higher than 1.25, need of G-CSF treatment, treatment interruption because of patient's refusal or toxicity. Because of possible confounding effect of radiotherapy associated to CMF, both toxicity and compliance were analysed not only by age subgroups but also according to presence or absence of concomitant radiotherapy.

Disease-free survival was defined as the time elapsed from surgery to the date of assessment of local or distant or contralateral breast cancer or the date of death for patients dying without disease recurrence. The Kaplan-Meier method was applied to draw disease-free survival curves.

Statistical significance of associations among variables was tested by the Fisher's exact test, or Wilcoxon-Mann Whitney test for naturally ordered variables (i.e. number of cycles, no. of day 8 omitted, grades of toxicity). Disease-free survival curves were compared by the Log-rank test. Analysis were done with S-PLUS (6.0 Professional Release 1, Insightful Corporation) and StatXact-5 (release 5.0.3, Cytel software Corporation).

## Results

From March 1991 to March 2002, 180 patients were identified, 80 aged 65 or older, and 100 older than 60 and younger than 65. The baseline characteristics of patients are reported in table 1. Overall, 87 patients received radiotherapy on the residual breast; among these, 21 patients, who received radiation therapy after the end of CMF, have been considered in the no radiotherapy subgroup for all subsequent analyses. Sixty-six patients receiving concomitant radiotherapy were equally distributed in the two age subgroups, 36 (36%) being younger than 65 and 30 (38%) being older.

Treatment toxicity scattered by age subgroups is reported in table 2. There was no toxic death. The only type of toxicity that significantly differed between the two age groups was febrile neutropenia that occurred in 3 cases among older patients and never in the younger group (p = 0.05). Among toxicities that did not show a statistically significant difference between the two groups, leukopenia, neutropenia, nausea, cardiac toxicity and thrombophlebitis tended to be more frequent or severe among elderlies, while mucositis tended to be more evident among younger patients. As shown in table 3, haematologic toxicity was adversely affected by concomitant radiotherapy, leukopenia, neutropenia, febrile neutropenia and fever being significantly worse among patients who received concomitant radiotherapy. As reported in table 4, incidence of grade 3–4 toxicity (either haematological or other or any type) did not vary according to age, while severe haematological toxicity was more frequent among patients who received concomitant radiotherapy (35% vs 8%, p < 0.0001); almost one half (47%) of the older patients receiving concomitant radiotherapy experienced grade 3–4 hematological toxicity.

Focusing on compliance, we found that CMF scheduling was modified (from days 1 and 8 every 4 weeks to day 1 every 3 weeks) because of toxicity in 7 patients (2 in the older and 5 in the younger group, p = 0.46); none of these patients did receive concomitant radiotherapy (p = 0.05). Distribution of compliance indices is summarized in table 5. Seventy-six percent of older patients received six cycles of chemotherapy as compared with 81% of those in the younger group. Day 8 of chemotherapy was withdrawn at least once in 36% and 31%, and 18% and 14% of the patients had more than 25% of cycle at reduced doses, in the older and younger groups, respectively. Treatment was administered without significant delay (i.e. 24 weeks without concomitant radiotherapy or 30 weeks with) in almost all the patients. Haematopoietic support with G-CSF was required by 18% and 9% of older and younger patients. Causes of treatment discontinuation were similar in the two groups, protocol completion accounting for more than three-fourths, and refusal and toxicity for about 10% each (p = 0.39). Eighty-eight percent of the 66 patients who received concomitant radiotherapy were able to receive 6 cycles of CMF and completed treatment according to the protocol, as compared with 74% of those not irradiated concomitantly; concomitant irradiation prolonged significantly duration of CMF in 5% of patients and produced a more frequent need of G-CSF. Overall, a weak compliance (i.e. the occurrence of at least one of the following events: less than 6 cycles received, more than 1 day 8 omission, dose reduction in more than 25% of cycles, relative duration higher than 1.25, use of G-CSF or treatment interruption because of toxicity or refusal) was more frequent among older patients (58% vs 46%, p = 0.02) and did not vary according to concomitant radiotherapy.

**Figure 1:**
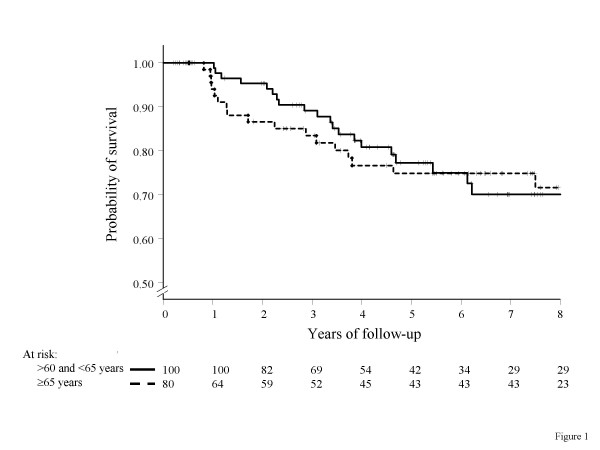
Kaplan-Meier estimated disease-free survival curves by age categories (solid line: >60 and <65 years; dashed line: ≥ 65 years; crosses indicate censoring)

As of March 2004, 41 patients had an event and disease-free survival at 5 and 8 years was 76% and 71%. As shown in figure 1, there was no statistically significant difference in disease-free survival in the two age groups (p = 0.84).

## Discussion

The present study was planned being aware that a selection bias, typical of retrospective data collection, could affect the results; namely, outside clinical trials, the therapeutic strategy applied in our Institution during the time period of interest for this study was prevalently conservative, foreseeing adjuvant chemotherapy in elderly patients when either a very high risk of relapse or a very strong patient's motivation existed, no severe or untreatable comorbid conditions being evident. Overall, we anticipated that, under such conditions and because of selection bias, our study should produce underestimation of toxicity and lack of compliance, this effect possibly being as more pronounced as older the patients because of the application of more restrictive criteria.

We selected for this study a consecutive series of 180 early breast cancer patients aged 60 to 73 that received, outside clinical trials, adjuvant CMF, with all drugs intravenously on days 1 and 8 every 4 weeks for 6 cycles; this schedule resulted as the most commonly used regimen in a survey circulated among the members of the Breast International Group [14]. Overall, 18% of the patients suffered grade 3–4 toxicity, and 47% had some problem of compliance, either because dosing and scheduling rules could not be followed appropriately, or because costly supportive drugs (G-CSF) were required. Both these figures are much higher than what we expected. In addition, problems were more frequent among the older subgroup (for compliance) and when radiotherapy was given concomitantly with CMF (for haematological toxicity). The sense of such finding is even strengthened by the fact that very old patients, i.e. those over 75, were not considered for treatment. Particularly, concomitant radiotherapy significantly prolonged duration of chemotherapy over the planned time in 5% of patients, haematological toxicity being the most reasonable cause, as suggested by a more frequent need of G-CSF. Paradoxically, because of more prolonged time and less intensive administration, the number of CMF cycles fared better among patients who received concomitant radiotherapy.

In a retrospective analysis of adjuvant CMF for elderly breast cancer patients enrolled in the International Breast Cancer Study Group (IBCSG) trial VII [15], 76 women 65 years of age or older, compared with 223 postmenopausal women younger than 65, showed higher grades of severe toxicity either considering any type (17% v 7%, respectively), or haematologic (9% v 5%, respectively). In addition, only 48% of the older patients as compared with 65% of the younger ones received at least 85% of the planned dose. Our findings in terms of toxicity and compliance are overall worse than in the IBCSG trial VII. The most reasonable explanation for this is the duration of CMF, that was planned for 3 cycles in the IBCSG trial and for 6 cycles in our routine practice; also, we cannot exclude effects of the type of schedule (all drugs intravenously versus cyclophosphamide orally on days 1–14) and the effect of the timing of radiotherapy, given at the end of chemotherapy in the IBCSG trial VII, while it was concomitant in 37% of the patients in our study. In addition, we hypothesize that selection bias, arising from typical exclusion criteria of randomized clinical trials, could also have lowered toxicity rate in the IBCSG trial VII. Anyway, the conclusion of the Authors that less toxic chemotherapy regimens are required for high-risk elderly patients is completely supported by our findings.

For elderly patients with breast cancer, adjuvant chemotherapy should be considered both when the tumor does not express hormonal receptors, because tamoxifen would be ineffective, and in many cases in which the tumor is receptor-positive, because several indirect, but reliable, evidences suggest that positive effects of chemotherapy and tamoxifen are not mutually exclusive but can sum up, at a given extent. Based on current knowledge, the problem could be structured saying that the choice of not giving adjuvant chemotherapy must be supported by valid reasons, inside the decision making process. Factors that can affect such decision are risk of relapse, life expectancy and patient's preferences. As for risk of relapse, several subsequent editions of the St.Gallen guidelines [4,6,16], widely accepted as a reasonable approach to the treatment of early breast cancer, rule out that patients with ER/PgR-negative can be considered at minimal risk. In addition, within endocrine responsive tumors, the minimal risk category is limited to a quite small group of patients. A large amount of elderly patients fall within prognostic categories at average or high risk of relapse. Extermann et al. [17] faced the problem of coupling the risk of cancer relapse or death with life expectancy in order to define a utility measure that can assist decision making. Based on current estimates of efficacy of adjuvant chemotherapy, they found that a risk of 12% at 10 years is the threshold value to produce an absolute 1% survival gain for a 75 years old patient, who has approximately 12 years of life expectancy, if not particularly sick; as for the risk of relapse, estimates show that older breast cancer patients can expect advantages from treatment fairly similar to that of younger patients, both for tamoxifen and for chemotherapy. Finally, patient's preferences are not an easy matter of study [18], and, particularly in the setting of adjuvant chemotherapy of elderly breast cancer patients, data are very poor. Some studies, mostly including patients younger than 70, have shown that age does not affect patient's preferences [19] and that most patients consider 6 months of adjuvant CMF chemotherapy worthwhile for relatively modest survival gains [20]. Furthermore, indirect data, regarding elderly cancer patients [21] with various cancer types, suggest that more than 70% of elderly patients are willing to receive effective chemotherapy even if toxicity is relevant; such data, however, should be confirmed in a particular clinical setting like that of adjuvant chemotherapy, where the treatment is given to reduce an invisible risk, rather than to cure a visible disease.

Anyway, while growing the scientific evidence that adjuvant chemotherapy should be considered for elderly patients with breast cancer at risk of relapse, several reports show that the use of adjuvant chemotherapy in clinical practice does dramatically decline with age. De Michele et al. showed that in a tertiary cancer centre the rate of women eligible for adjuvant chemotherapy according to international guidelines who were actually offered chemotherapy dramatically decreased from 77%, among those aged 61 to 69, to 23%, among those aged 70 ore more [22]. Mandelblatt et al., from the Outcomes and Preferences for Treatment in Older Women Nationwide Study (OPTIONS) project, reported that, within a cohort of 718 patients, treated at public hospitals in several US states between 1995 and 1997, only 7% of those aged 67 to 79 years received adjuvant chemotherapy [23]. Woodard et al., after adjusting for a number of possible confounders, found that the odds of not receiving adjuvant chemotherapy for patients older than 65 is 62 times higher than that of patients younger than 50, and, even if the analysis is limited to ER-negative cases (i.e. those not amenable to hormonal adjuvant treatment), an odds of 7 is calculated [24].

There can be a lot of reasons for the discrepancy between guidelines or clinical trials evidences and practical application. One we believe is very important, together with other Authors [25], is the paucity of clinical trials specifically addressing adjuvant chemotherapy of elderly patients, because it is conceivable that lack of reliable evidences does not encourage physicians to suggest treatments that are potentially toxic and dangerous [26]. The consequences of such phenomenon are relevant, considering that sensible differences in patients outcome can be produced by varying treatment approaches [27].

## Conclusion

In conclusion, the present data, together with other retrospective evidences, strongly highlight the need of clinical trials of adjuvant chemotherapy specific for elderly early breast cancer patients, possibly looking for drugs or schemes that have shown in previous feasibility studies a favourable profile of safety. In this light, in a randomised clinical trial open to early breast cancer patients aged 65 to 80 with an average/high risk of relapse according to St.Gallen criteria, we are actually comparing CMF with weekly docetaxel, a treatment scheme that has shown sufficient activity and excellent tolerability in a phase II study conducted in elderly advanced breast cancer patients [28].

## Competing interests

The author(s) declare that they have no competing interests.

## Authors' contributions

EDM, FP and AdM conceived of the study, participated in its design and coordination and drafted the manuscript;

AG, CP, GA, VL, GL, FN, ER, GD'A, IC, MR, BM, ME and MDM treated the patients, collected the clinical data useful for the analysis and revised the article critically for important intellectual content;

All authors read and approved the final manuscript.

## Pre-publication history

The pre-publication history for this paper can be accessed here:



## Figures and Tables

**Table 1:** Characteristics of Patients (N = 180)

Median age (range), yrs	64 (60–73)
Age category, n (%)	
>60 and < 65 years	100 (56)
≥ 65 years	80 (44)
pT-category, n (%)	
pT1	73 (41)
pT2	91 (51)
pT3	5 (3)
pT4	3 (2)
unknown	8 (4)

pN-category, n (%)	
pN0	66 (37)
pN1	97 (54)
pN2	10 (6)
unknown	7 (4)

Receptor status, n (%)	
ER or PgR positive	82 (46)
Negative	78 (43)
Unknown	20 (11)

Histotype, n (%)	
Ductal	132 (73)
Lobular	33 (18)
Other	15 (8)

Histologic grade, n (%)	
G1	5 (3)
G2	53 (29)
G3	96 (53)
Unknown	26 (14)

Concomitant radiotherapy, n (%)	
No	114 (63)
Yes	66 (37)

**Table 2:** Worst degree of toxicity by age group (% of patients)

Type of toxicity	>60 and <65 years (n = 100)					≥ 65 years (n = 80)					P*
		
	0	1	2	3	4	0	1	2	3	4	
Anemia	81	10	8	0	1	86	6	4	4	0	*0.39*
Leukopenia	54	15	19	7	5	45	11	26	11	6	*0.13*
Neutropenia	73	10	8	5	4	68	9	7	10	6	*0.32*
Febrile Neutropenia	100	-	-	0	0	96	-	-	4	0	*0.05*
Infection	97	0	1	2	0	95	0	2.5	2.5	0	*0.50*
Fever	98	0	2	0	0	98	2	0	0	0	*0.83*
Thrombocytopenia	90	7	2	0	1	91	4	1	3	1	*0.83*
Bleeding	98	1	0	1	0	100	0	0	0	0	*0.21*
Nausea	81	8	6	5	0	69	11	12	8	0	*0.06*
Vomiting	71	5	11	13	0	69	9	16	6	0	*0.98*
Mucositis	84	5	8	3	0	92	1	5	1	0	*0.09*
Skin	99	1	0	0	0	98	0	2	0	0	*0.43*
Diarrhea	96	1	0	3	0	96	3	1	0	0	*0.91*
Constipation	99	0	0	1	0	98	1	1	0	0	*0.45*
Hepatic	88	3	7	2	0	90	6	4	0	0	*0.60*
Cardiac	100	0	0	0	0	96	0	2.5	1	0	*0.09*
Fatigue	96	1	3	0	0	95	0	2.5	2.5	0	*0.72*
Pulmonary	100	0	0	0	0	99	1	0	0	0	*0.27*
Abdominal pain	99	0	0	1	0	99	0	0	1	0	*0.88*
Chest pain	99	0	0	1	0	100	0	0	0	0	*0.37*
Heartburn	99	0	1	0	0	98	0	2	0	0	*0.44*
Headache	99	0	0	1	0	100	0	0	0	0	*0.38*
Confusion	99	1	0	0	0	100	0	0	0	0	*0.38*
Depression	100	0	0	0	0	99	0	0	1	0	*0.27*
Thrombophlebitis	97	0	0	3	0	94	0	0	6	0	*0.30*
Dysuria	99	0	0	1	0	99	0	0	1	0	*0.88*
Allergy	99	0	0	1	0	100	0	0	0	0	*0.37*
Weight gain	100	0	0	0	0	99	0	1	0	0	*0.27*
Weight loss	99	0	1	0	0	99	1	0	0	0	*0.89*

* Wilcoxon-Mann Whitney test

**Table 3:** Worst degree of hematological toxicity by radiotherapy (% of patients)

Type of toxicity	No (n = 114)					Yes (n = 66)					P*
		
	0	1	2	3	4	0	1	2	3	4	
Anemia	87	6	4	3	0	77	12	9	0	1.5	*0.12*
Leukopenia	58	15	22	3	3	36	11	23	20	11	*0.0001*
Neutropenia	82	8	6	2	2	50	12	11	17	11	*<0.0001*
Febrile Neutropenia	100	0	0	0	0	95	0	0	5	0	*0.05*
Infection	96	0	2	2	0	95	0	2	3	0	*0.89*
Fever	100	0	0	0	0	94	6	0	0	0	*0.02*
Thrombocytopenia	90	6	2	1	1	91	4.5	1.5	1.5	1.5	*0.96*
Bleeding	99	0	0	1	0	98	2	0	0	0	*1.00*

* Wilcoxon-Mann Whitney test

**Table 4:** Summary of toxicity by age, radiotherapy and combined subgroups expressed as row percentages)

	**Haematologic**			**Other**			**Any**		
			
**Subgroup**	**G0-2**	**G3-4**	p*	**G0-2**	**G3-4**	p*	**G0-2**	**G3-4**	p*
**Age**			0.17			0.49			1.00
>60 and <65 years (*n = 100*)	86%	14%		72%	28%		62%	38%	
≥ 65 years (*n = 80*)	78%	22%		78%	22%		61%	39%	

**Radiotherapy**			<0.0001			0.21			0.27
No (*n = 114*)	92%	8%		71%	29%		65%	35%	
Yes (*n = 66*)	65%	35%		80%	20%		56%	44%	

**Age and radiotherapy**			<0.0001			0.42			0.08
>60 and < 65, no RT (*n = 64*)	92%	8%		67%	33%		59%	41%	
≥ 65, no RT (*n = 50*)	92%	8%		76%	24%		72%	28%	
>60 and < 65, RT (*n = 36*)	75%	25%		81%	19%		67%	33%	
≥ 65, RT (*n = 30*)	53%	47%		80%	20%		43%	57%	

G = grade; RT = radiotherapy.

**Table 5:** Pattern of compliance to chemotherapy by age and radiotherapy

Variable	Age			Radiotherapy		
		
	>60 and < 65 (*n = 100*)	≥ 65 (*n = 80*)	P^§^	No* (*n = 114*)	Yes (*n = 66*)	P^§^
N. of cycles administered, no. (%)			0.42			0.01
6 cycles	81 (81)	61 (76)		84 (74)	58 (88)	
5 cycles	5 (5)	4 (5)		4 (3)	5 (8)	
4 cycles	4 (4)	4 (5)		6 (5)	2 (3)	
3 cycles	5 (5)	6 (8)		10 (9)	1 (1)	
2 cycles	4 (4)	4 (5)		8 (7)	0	
1 cycle	1 (1)	1 (1)		2 (2)	0	

N. of day 8 omitted, no. (%)			0.37			0.86
0	69 (69)	51 (64)		77 (68)	43 (65)	
1	23 (23)	18 (22)		24 (21)	17 (26)	
2	5 (5)	8 (10)		9 (8)	4 (6)	
3	3 (3)	3 (4)		4 (3)	2 (3)	

Rate of cycles at reduced dose, no. (%)			0.54			0.83
≤ 25%	86 (86)	66 (82)		97 (85)	55 (83)	
>25%	14 (14)	14 (18)		17 (15)	11 (17)	

Actual/planned duration, no. (%)			0.59			0.05
≤ 1.25	99 (99)	78 (98)		114 (100)	63 (95)	
>1.25	1 (1)	2 (2)		0	3 (5)	

G-CSF utilization, no. (%)			0.12		<.0001	
No	91 (91)	66 (82)		109 (96)	48 (73)	
Yes	9 (9)	14 (18)		5 (4)	18 (27)	

Treatment discontinuation, no. (%)			0.52			0.10
protocol completion	81 (81)	61 (76)		84 (74)	58 (88)	
patient's refusal	6 (6)	9 (11)		12 (11)	3 (4)	
treatment toxicity	11 (11)	7 (9)		13 (11)	5 (8)	
other	2 (2)	3 (4)		5 (4)	0	

Low compliance^†^, no. (%)			0.02			0.88
No	61 (61)	34 (42)		61 (54)	34 (52)	
yes	39 (39)	46 (58)		53 (46)	32 (48)	

* including 21 patients who received radiotherapy after the end of chemotherapy

§ Fisher exact test or Wilcoxon-Mann Whitney test for naturally ordered variables)

† at least one of the following features: less than 6 cycles administered, withdrawal of more than one day-8 treatment, dose reduction in more than 25% of cycles, relative duration higher than 1.25, use of G-CSF treatment, treatment interruption because of patient's refusal or toxicity
